# *Lactobacillus crispatus* Dominant Vaginal Microbiome Is Associated with Inhibitory Activity of Female Genital Tract Secretions against *Escherichia coli*

**DOI:** 10.1371/journal.pone.0096659

**Published:** 2014-05-07

**Authors:** Jeny P. Ghartey, Benjamin C. Smith, Zigui Chen, Niall Buckley, Yungtai Lo, Adam J. Ratner, Betsy C. Herold, Robert D. Burk

**Affiliations:** 1 Department of Obstetrics & Gynecology and Women’s Health, Albert Einstein College of Medicine, Bronx, New York, United States of America; 2 Department of Pediatrics, Albert Einstein College of Medicine, Bronx, New York, United States of America; 3 Departments of Epidemiology and Population Health, Albert Einstein College of Medicine, Bronx, New York, United States of America; 4 Department of Pediatrics, Columbia University College of Physicians and Surgeons, New York, New York, United States of America; 5 Department of Microbiology-Immunology, Albert Einstein College of Medicine, Bronx, New York, United States of America; INRA Clermont-Ferrand Research Center, France

## Abstract

**Objective:**

Female genital tract secretions inhibit *E. coli* ex vivo and the activity may prevent colonization and provide a biomarker of a healthy microbiome. We hypothesized that high *E. coli* inhibitory activity would be associated with a *Lactobacillus crispatus* and/or *jensenii* dominant microbiome and differ from that of women with low inhibitory activity.

**Study Design:**

Vaginal swab cell pellets from 20 samples previously obtained in a cross-sectional study of near-term pregnant and non-pregnant healthy women were selected based on having high (>90% inhibition) or low (<20% inhibition) anti-*E. coli* activity. The V6 region of the 16S ribosomal RNA gene was amplified and sequenced using the Illumina HiSeq 2000 platform. Filtered culture supernatants from *Lactobacillus crispatus, Lactobacillus iners,* and *Gardnerella vaginalis* were also assayed for *E. coli* inhibitory activity.

**Results:**

Sixteen samples (10 with high and 6 with low activity) yielded evaluable microbiome data. There was no difference in the predominant microbiome species in pregnant compared to non-pregnant women (n = 8 each). However, there were significant differences between women with high compared to low *E. coli* inhibitory activity. High activity was associated with a predominance of *L. crispatus* (p<0.007) and culture supernatants from *L. crispatus* exhibited greater *E. coli* inhibitory activity compared to supernatants obtained from *L. iners* or *G. vaginalis.* Notably, the *E. coli* inhibitory activity varied among different strains of *L. crispatus.*

**Conclusion:**

Microbiome communities with abundant *L. crispatus* likely contribute to the *E. coli* inhibitory activity of vaginal secretions and efforts to promote this environment may prevent *E. coli* colonization and related sequelae including preterm birth.

## Introduction

Preterm birth is a major health problem affecting 12.3% of live births and is a major cause of neonatal mortality [1], [2]. *Escherichia coli* (*E. coli*) has been associated with preterm birth [3], is among the leading cause of neonatal sepsis in extremely low birth weight neonates [4], and is the leading cause of early-onset neonatal sepsis and meningitis [5]. Similarly, bacterial vaginosis (BV), a dysbiotic condition characterized by the replacement of a *Lactobacillus crispatus (L. crispatus)* predominant microbiome with anaerobic species [6], [7], has also been linked to an increased risk of preterm birth, although treatment with antibiotics in late pregnancy was ineffective [8]–[10]. These findings indicate that the vaginal microbiome plays an important role in adverse pregnancy outcomes.

Recent studies suggest that the vaginal microbiome may be linked to soluble mucosal defense. Specifically, genital tract secretions have been consistently shown to possess *in vitro* inhibitory activity against *E. coli* [11]–[15]. This activity, which may reflect contributions from the host innate immune mediators such as defensins as well as from microbiota, may be critical during pregnancy and prevent dysbiotic vaginal colonization and ascending infection. Earlier studies showed that the *E. coli* inhibitory activity was reduced among non-pregnant women with BV and was restored following successful treatment with metronidazole [11]. In a recent cross sectional study, genital tract secretions obtained by vaginal swabs from near term healthy pregnant women were found to have significantly higher inhibitory activity against *E. coli* that was inversely correlated with *E. coli* vaginal colonization [12]. A separate study using cervicovaginal lavage (CVL) samples from healthy non-pregnant women suggested that the inhibitory activity may be mediated, at least in part, by soluble proteins secreted by lactobacilli [15]. Using biochemical techniques including mass spectrometry, four *Lactobacillus* proteins (three originally described as proteins of *L. crispatus* and one of *L. jensenii*) were present exclusively in CVL samples with high (>90% inhibitory activity), but not in samples with low activity (<20%). These proteins included the S-layer protein, a bacterial surface layer protein, and a cell separation protein for *L. crispatus* and adhesion exoprotein for *L. jensenii* [15].

Building on this background, the current study was designed to further evaluate the link between *E. coli* inhibitory activity and the vaginal microbiome and to test the hypothesis that high *E. coli* inhibitory activity would be associated with healthy *Lactobacillus species* dominant microbiome whereas low activity would be associated with a more diverse microbiome. Utilizing samples previously obtained from healthy near term pregnant and non-pregnant women, we also tested the hypothesis that pregnancy would alter the microbiome, as suggested in a prior study [16]. A subset of samples were selected from 10 pregnant and 10 non-pregnant women and within each cohort, five were selected for high inhibitory activity (defined as >90% reduction in number of *E. coli* colony forming units [cfu]) and five with low activity (defined as <20% reduction in cfu).

## Materials and Methods

### Participants and Sample Collection

The parent study was described previously in detail [12]. Briefly, healthy pregnant women were recruited between 35 and 37 weeks of gestation and healthy non-pregnant were recruited during a routine gynecologic visit. Vaginal swabs were collected to measure soluble immune mediators and endogenous *E. coli* inhibitory activity. Following approval from the Montefiore Medical Center Internal Review Board, all women gave written informed consent. For this sub-study, vaginal swab pellets from 10 pregnant women between 35 and 37 weeks of gestation and 10 healthy non-pregnant women (5 with *E. coli* inhibitory activity >90% and 5 with activity <20% within each group) were selected and analyzed in a blinded manner for the vaginal microbiome. None of the participants had clinical BV using Amsel’s criteria [17].

Vaginal swabs were placed in a 1.5 mL sterile eppendorf tube that was pre-filled with 0.5 mL of sterile normal saline, placed on ice, and processed within 6 hours of collection. The sample was vortexed and clarified by centrifugation at 2000 rpm for 7 minutes at 4°C and the cell pellets were re-suspended in 200 µL of phosphate buffered solution (PBS) and stored at −80°C until used for the microbiome analysis. The supernatants were divided into aliquots and used to measure *E. coli* inhibitory activity as well as concentrations of immune mediators [12]. The Internal Review Board of Albert Einstein College of Medicine approved the parent study; all participants signed informed consent and only subjects who agreed that samples could be used for future studies were included.

### DNA Extraction and Amplification

Relatively low-cycle amplification and next generation sequencing (NGS) on the Illumina HiSeq2000 platform, in conjunction with pplacer software was used to analyze the bacterial composition of vaginal samples. DNA for sequencing was extracted from pelleted cells by incubating 150 µl of sample in 250 µl of proteinase K digestion cocktail containing 1% sodium laureth-12 sulfate at 55°C for 2 hours heated to 95°×10 minutes and then the DNA was precipitated in a 0.825 M ammonium acetate/ethanol (AAE) solution, pelleted by centrifugation and re-suspended in TE (10 mM Tris, pH 8.0, 0.1 mM EDTA), as described previously [18]–[20]. The V6 region of 16S rRNA genes were amplified in an ABI 9700 Thermal Cycler (Life, Carlsbad, CA) using HotStart-IT FideliTaq DNA Polymerase (Affymetrix, Santa Clara, CA) and 50 ng of template DNA in a total volume of 50 µl. Reaction parameters included initial denaturation at 94°C for 2 min, followed with 30 cycles of denaturation at 94°C for 30 seconds, annealing at 60°C for 30 seconds, and extension at 68°C for 30 seconds; and a final extension at 68°C for 5 min. Sense target primers contained unique 8-bp Hamming DNA barcodes [21], which allowed for the identification of reads from each sample. Successfully amplified DNA from all samples was pooled, purified and isolated using gel electrophoresis and electroelution. Following quality control and library preparation, DNA was sequenced on an Illumina HiSeq 2000 using a paired-end protocol yielding 100 base pairs of sequence in each direction. Raw sequences were joined (using eautils’ fastq-join), processed to remove chimeras (using uchime), and low quality reads and nucleotides, and assigned to their sample of origin by demultiplexing (using mubiomics). Demultiplexed reads were aligned to the bacterial reference library - a vaginal microbiome reference library available from http://microbiome.fhcrc.org/apps/refpkg/, containing 633 sequences, representing 138 bacterial taxa, using pyNAST. The aligned sequences were then mapped to their bacteria of origin using the classification algorithm pplacer [22] and visualized using R-scripts that were developed at Einstein [18] (see Statistical Analyses section).

### *E. coli* Inhibitory Activity

Spent culture supernatants (SCS) from *L. crispatus* (ATCC 33197, M35, SJ-3C, and 60), *L. iners* (DSM 13335, UPII 60B-BEI HM-131, and 143D-BEI HM-126), and *G. vaginalis* (ATCC 49145, 14018) were prepared by centrifuging overnight cultures of bacteria at 2000 g for 15 min at room temperature and then filtering the supernatant to remove bacteria and particulate matter (0.22 µm syringe filter). *L. crispatus* strains were grown in MRS broth; *L. iners* in 1% proteose peptone, 1% beef extract, 0.5% yeast extract, 86 mM NaCl, 0.8 mM MgSO_4_, 0.3 mM MnSO_4_, 11.5 mM K_2_HPO_4_, 10% fetal bovine serum, and 2% glucose; and *G. vaginalis* in brain heart infusion broth supplemented with 10% FBS and 5% Fildes enrichment. The SCS from each of these cultures and their respective growth media were serially diluted in normal saline and then incubated with *E. coli* (ATCC strain 43827) (∼10^9^ cfu/mL) for 2 h at 37°C. The mixtures were then further diluted in saline (to yield 800–1000 *E. coli* colonies on control plates) and plated on agar enriched with trypticase soy broth [15]. Colonies were counted using ImageQuant TL v2005 after an overnight incubation at 37°C. To control for differences in cfu of the *L. crispatus*, *L. iners* or *G. vaginalis* cultures, the inhibitory activity was normalized to lowest bacterial yield in a *post-hoc* analysis or, alternatively, the SCS were diluted based on different yields prior to incubating them with *E. coli*. All samples were tested in duplicate and the percentage inhibition was determined relative to the colonies formed on culture media control plates.

### Statistical Analyses

*E. coli* inhibitory activity was dichotomized as >90% (high) and <20% (low). Categorical variables were compared between groups by Chi-square or Fisher’s exact test. Continuous variables were compared by the Student t test or the Mann-Whitney U test, depending on the distribution of the data. Clinical data were analyzed using STATA (v11.0; StataCorp, Inc., College Station, TX). All plotting and statistical comparisons for NGS were performed in R v2.12.2 using a script developed in-house (available upon request). The pairwise Kantorovich-Rubinstein (KR) distances (equivalent to the weighted UniFrac distance [23] between samples were calculated with *p* = 1 and normalized with respect to the diameter of the reference tree. Principal component analysis was performed to determine differences between pregnancy and the microbiome and dichotomized *E. coli* inhibitory activity and the microbiome. Using a conservative approach, the PERMANOVA analysis was performed on the Kantorovich-Rubinstein (KR) distances between sample microbiomes, with pregnancy and *E. coli* inhibitory activity as the factors, respectively. Fisher’s exact tests were performed with pregnancy status and *E. coli* inhibitory activity as binary factors against the dominant bacterial taxon. Shannon and Simpson diversity indices were calculated to determine significant differences in diversity between pregnant and non-pregnant samples and samples with high and low *E. coli* inhibitory activity. To ensure that sufficient sampling of the microbiome had occurred, a rarefaction analysis was performed. Bacterial taxa were grouped to reflect healthy lactobacilli (group 1), intermediate bacteria (at times associated with BV diagnosed by Nugent’s score [24], group 2) and pathogenic bacteria (groups 3 and 4): Group 1: *L. crispatus, L. jensenii,* and *L. gasseri*, Group 2: *L. iners*, Group 3: *G. vaginalis, Sneathia Sanguinegens*, BVAB1, BVAB2, *Megasphaera, Prevotella bivia, Prevotella melaninogenica, Prevotella* genogroup 1 and genogroup 2, and *Atopobium vaginae*, and Group 4: *Streptococcus anginosus* and *Staphylococcus hominis*. Principal component analyses were performed using Group 1, Group 3, and Group 4 to reduce the dimensions of the data. Spearman correlation coefficients (SCC) were used to examine correlations between bacterial groups and soluble immune mediators measured in the parent study [12]. The following mediators were included in the analysis: secretory leukocyte protease inhibitor (SLPI), human neutrophil peptide 1–3 (HNP1–3), human beta defensin (HBD)-1, HBD-2, and HBD-3, and cytokines/chemokines including interleukin (IL)-1α, IL-1β, IL-1 receptor antagonist (IL-1ra), IL-6, IL-8, macrophage inhibitory protein (MIP)-1α, MIP-1β, and regulated on activation, normal T-cell expressed and secreted (RANTES). All tests were two-sided with p value of <0.05 considered statistically significant.

## Results

### Description of Participants

Sixteen of the 20 subjects had paired-end reads that were sufficient to characterize the microbiome. The characteristics of these subjects are summarized in Table 1 grouped according to *E. coli* inhibitory activity (10 with high activity and 6 with low activity). The two groups did not differ with respect to age, race, number of pregnant women, current smoking status, history of prior sexually transmitted infections (STI), contraceptive use, method of vaginal swab collection (physician or self-collected), or vaginal wall pH (Table 1).

**Table 1:** Demographic and clinical characteristics of women with low and high *E. coli* inhibitory activity.

	Low *E. coli* inhibitoryactivity^a^ (n = 6)	High *E. coli* inhibitoryactivity^b^ (n = 10)	p-value^c^
Age, median (range)	24 (22–41)	26.5 (22–31)	0.30
Race, n (%)			0.70
White	5 (83)	8 (80)	
Black	1 (17)	1 (10)	
Other	0	1 (10)	
Pregnant			1.0
No	3 (50)	5 (50)	
Yes	3 (50)	5 (50)	
Current Smoker, n (%)	1 (17)	0	0.38
History of genital herpes, n (%)	0	2 (20)	0.50
History of chlamydia, n (%)	1 (17)	1 (10)	1.0
History of gonorrhea, n (%)	0	0	1.0
History of genital warts, n (%)	1 (17)	0	0.38
Contraception, n (%)			1.0
None	4 (67)	9 (90)	
Barrier Methods	0	0	
Oral contraceptive pills	0	1 (10)	
Intra-vaginal ring	0	0	
Progesterone injectable	1 (17)	0	
Progestin-containing IUD	1 (17)	0	
Lateral vaginal wall pH, median (range)	4.9 (4.6–5.2)	4.6 (4.2–5.5)	0.31
Method of swab collection, n (%)			0.45
Physician-collection	3 (50)	5 (50)	
Observed self-collection	3 (50)	5 (50)	

a >90% inhibition.

b <20% inhibition.

c p-value<0.05 considered significant.

### Microbiome Analysis

NGS produced 9,111,237 high quality, non-chimeric, joined, paired-end reads, of which 8,946,862 were successfully assigned a bacterial identity. Those that could not be aligned (and therefore assigned) were of non-bacterial origin. Less than 2% of the reads were determined to be chimeric and were therefore removed. The short reads were submitted to the NCBI Short-Read Archive (Submission ID: SRP034665).

**Figure 1:**
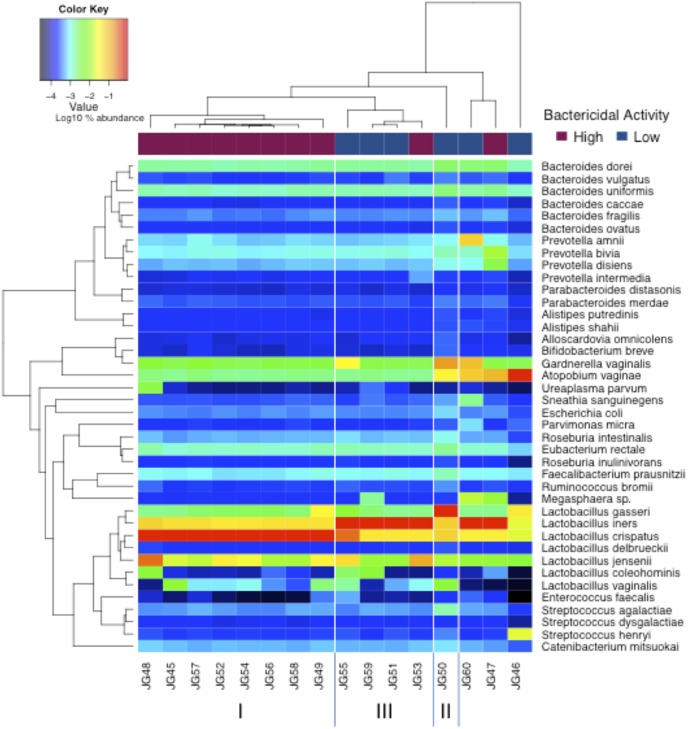
High *E. coli* inhibitory activity is associated with *L. crispatus* predominant microbiome. Heat Map of community compositions and proportional abundances of bacteria. The first row of colored tiles indicates the level of *E. coli* inhibitory activity (claret = >90% inhibition and blue <20% inhibition). Each subsequent row represents the bacterial taxon and its proportional abundance (on a base 10 logarithmic scale). A taxon is only shown if ≥217 reads were assigned there for any sample (corresponding to retaining all reads above the 90^th^ percentile). The Roman numerals at the bottom of the figure correspond to vaginal microbiome groups as reported by Ravel et al. The last 3 samples have not been classified do to limited sample size in this region of the dendogram.

Community compositions and proportional abundances of bacteria are shown in the heat map of Figure 1. All but one of the samples (JG46) was dominated by *Lact*o*bacillus* species including *L. crispatus* and *L. iners*, which is consistent with the absence of clinical BV in the cohorts. Most strikingly, the majority of samples with high *E. coli* inhibitory activity were dominated by *L. crispatus*, while those with low activity were dominated by *L. iners.* The two exceptions were JG46 and JG50, which had a microbiome dominated by *Atopobium vaginae* and *Lactobacillus gasseri*, respectively; both samples were in the low *E. coli* inhibitory activity cohort. The microbiome communities found in the samples clustered into three groups: *L. crispatus, L. iners,* and *L. gasseri* and are consistent with the community types found by Ravel et al. [6] A rarefaction plot is a standard analysis used to evaluate whether sufficient reads have been tested to detect species richness. If the gradient of the rarefaction curve decreases sharply after some level of species richness, it is a good indication that increasing the read depth would discover only the very rarest of species. Due to the low gradient of the curves of the rarefaction analysis at the read depth at which the clinical samples were sequenced, only the rarest of bacteria are likely to have been missed from our analyses (data not shown).

**Figure 2:**
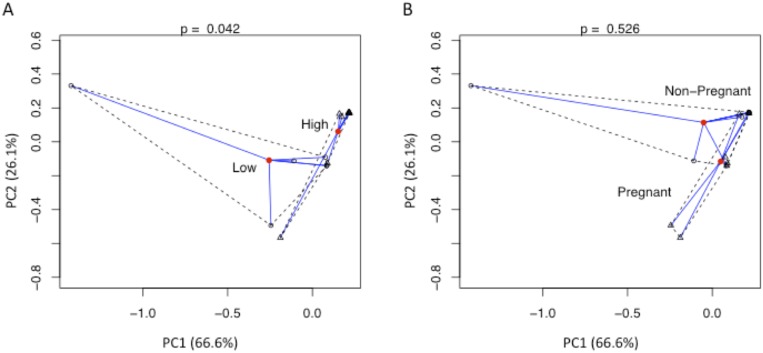
The microbiome associated with high *E. coli* inhibitory activity is significantly different than the microbiome in women with low activity (p = 0.042). The pairwise Kantorovich-Rubinstein (KR) distances between sample microbiomes were calculated and a PERMANOVA principal component analysis was performed with *E. coli* inhibitory activity (A) and pregnancy (B) as the factors. The two axes represent the first two principal components of the pairwise KR distance matrix. The red point demarks the group centroid, while the black open points represent the sample coordinates in the first two principal components. The p-value test statistic is displayed at the top of each plot area, indicating the statistical significance of the difference in variances when samples were grouped according to the factor (i.e., *E. coli* inhibitory activity or pregnancy). The Eigen values, or the amount of variation in the data accounted for by each principal component, are found in parentheses adjacent to PC1 and PC2.

High *E. coli* inhibitory activity was predictive of an *L. crispatus* dominated microbiome (Fisher’s exact test, p value = 0.007), while low activity was not significantly associated with any dominant bacterial taxon. Furthermore, the vaginal microbiome in women with high *E. coli* inhibitory activity were significantly different than women with low activity by principal component analysis (p = 0.001, Figure 2A). However, there were no differences between the pregnant and non-pregnant women (Figure 2B) and no association was found between pregnancy and any of the dominant bacterial taxa. Furthermore, we found no significant association between age, race, vaginal wall pH and *L. crispatus* predominance. There was also no difference in bacterial diversity between pregnant and non-pregnant women or between those with high or low *E. coli* inhibitory activity using Shannon and Simpson diversity indices (not shown).

In the parent study, the concentrations of protein and a subset of cytokines, chemokines, defensins and antimicrobial peptides were measured in the vaginal swab supernatants and SLPI, HBD-1, HBD-2, IL-1β, IL-6, and IL-8 correlated modestly with *E. coli* inhibitory activity among non-pregnant, but not pregnant women [12]. To assess whether any of these mediators were associated with the bacterial groups, Spearman correlation coefficients were measured. None of the bacterial groups correlated with the soluble immune mediators.

### Spent Culture Supernatants from *L. crispatus* have Significant *E. coli* Inhibitory Activity

**Figure 3:**
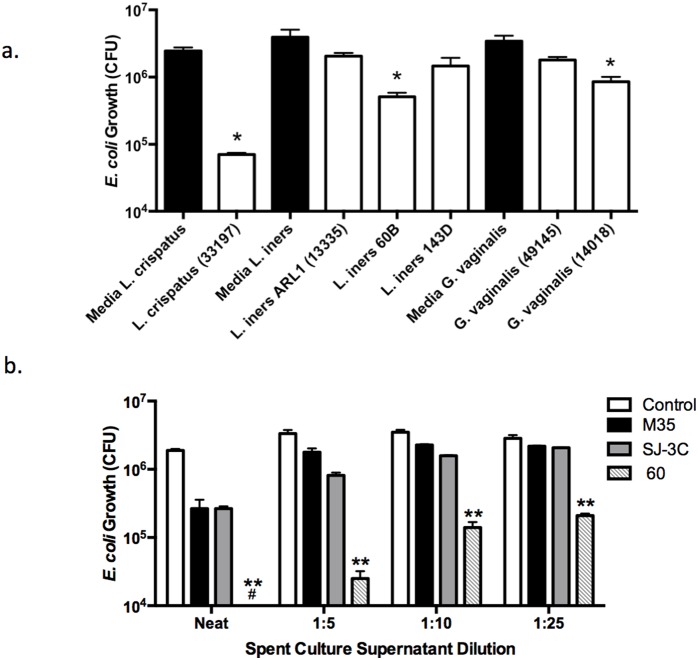
*L. crispatus* culture supernatants inhibit *E. coli.* (a) Bar graphs depicting the *E. coli* cfu/ml after overnight incubation with 1∶10 dilution in normal saline of culture supernatants obtained from the indicated bacterial species or respective control media. The results were adjusted for differences in colony counts of the bacteria from which SCS were obtained and are presented as mean ± SD obtained from 3 independent experiments. (b) The spent culture supernatants obtained from three additional strains of *L. crispatus* (M35, SJ-3C, and 60) were normalized by diluting in culture media to that of the lowest growth (2×10^7^ cfu/ml) and then serial dilutions (neat, 1∶5, 1∶10 and 1∶25) were mixed with *E. coli* and tested for inhibitory activity. Results are mean ± SD from duplicate plates. The number symbol indicates that no bacterial colonies were observed on plates after incubation with undiluted *L. crispatus* 60 SCS. The asterisks represent a significant reduction in *E. coli* cfu relative to its control growth media (p<0.01).

To gain further insight into the link between the microbiome composition and *E. coli* inhibitory activity, SCS from *L. crispatus*, *L. iners*, and *G. vaginalis* were tested for the ability to inhibit *E. coli* relative to the growth media for each bacteria. The bacterial yields of the cultures used to produce the SCS were as follows: *L. crispatus* 33197: 2.5×10^9^ cfu/mL; *L. iners* ARL1 (13335): 4.2×10^8^ cfu/mL; *L. iners* 60B: 1.0×10^9^ cfu/mL; *L. iners* 143D: 6.5×10^8^ cfu/mL; *G. vaginalis* 49145: 4.5×10^8^ cfu/mL; *G. vaginalis* 14018∶1.0×10^9^ cfu/mL. When the SCS were diluted 1∶10 prior to mixing with *E. coli, L. crispatus* 33197 showed the greatest magnitude of inhibitory activity and reduced the *E. coli* cfu by greater than 1 log after adjusting for differences in bacterial yields, although SCS from *L. iners* 60B *and G. vaginalis* 14018 also showed significant inhibitory activity (p<0.05) (Figure 3a). To further explore the inhibitory activity of *L. crispatus*, we tested serial dilutions of 3 additional strains (after diluting the SCS to adjust for differences in bacterial yields as follows: *L. crispatus* M35: 4.1×10^8^ cfu/mL; *L. crispatus* SJ-3C: 1.3×10^8^ cfu/mL; and *L. crispatus* 60: 2×10^7^ cfu/mL). *L. crispatus* 60 displayed the most potent activity and retained significant activity even after dilution of 1∶25 (Figure 3b).

## Discussion

The current study demonstrates that a *L. crispatus* dominant vaginal microbiome community is associated with *E. coli* inhibitory activity and supports the contention that the microbiome contributes to host defense. This notion is further supported by the finding that culture supernatants from *L. crispatus* had the most potent inhibitory activity, although the activity varied between different strains. The results are consistent with prior observations that *E. coli* inhibitory activity is reduced in the setting of BV, a dysbiotic condition associated with loss of *L. crispatus* [6], [7] and with a small proteomic study showing that *Lactobacillus* proteins (three originally described as proteins of *L. crispatus* and one of *L. jensenii*) were present exclusively in CVL samples with high but not in samples with low *E. coli* inhibitory activity [15]. While the microbiome from women with high *E. coli* inhibitory activity was dominated by *L. crispatus,* women with low activity (even in the absence of clinical BV) had a trend towards *L. iners* dominance. However, we did not observe any significant increase in bacterial diversity in samples from women with low *E. coli* inhibitory activity. It is possible that the other common healthy *Lactobacillus species* such as *L. jensenii*, also contribute to *E. coli* inhibitory activity. *L. jensenii* was one of the more prevalent bacteria in the women with high activity (Figure 1).

There were no differences detected in the vaginal microbiome between healthy near term pregnant and non-pregnant women. These findings are consistent with one other study in which the microbiome exhibited less diversity and richness in pregnant women sampled between 18–32 weeks gestation, but returned to the non-pregnant community structure in late gestation (>32 weeks) [16]. Both studies focused on healthy women, the majority of whom delivered at term. Larger studies in women at risk for preterm birth are needed to determine whether changes in the vaginal microbiome and/or *E. coli* inhibitory activity will provide a biomarker of risk for adverse outcomes including *E. coli* colonization and associated sequelae (e.g. chorioamnionitis and neonatal sepsis).

The potential utility of *E. coli* inhibitory activity as a biomarker of mucosal health, however, may differ in populations where lactobacilli species are not the dominant microflora. This notion is supported by two small sub-studies of African women who were at high-risk for HIV acquisition [25]. In the latter studies, having higher *E. coli* inhibitory activity was associated with an increased risk of HIV acquisition and, in one study, with a higher viral set point [26]. However, the participants in these studies had relatively high Nugent scores and lower median *E. coli* inhibitory activity (50% inhibition) compared to that observed in the healthy U.S cohorts (>70% inhibition). Moreover, in the HIV seroconverters, *E. coli* inhibitory activity correlated with the concentrations of several pro-inflammatory cytokines and chemokines, suggesting that the activity may be a biomarker of inflammation in high-risk women. In contrast, in the current study, there were no significant correlations between concentrations of mucosal immune mediators and the bacterial groups further indicating that perhaps host immune factors contribute little to this antimicrobial activity in populations where the microbiome is typically dominated by protective *Lactobacillus* species. Thus, in populations where there is a relative paucity of protective lactobacilli (e.g. high risk African cohorts), *E. coli* inhibitory activity may be more influenced by inflammatory molecules and serve as a biomarker of HIV risk, whereas in populations where *L. crispatus* is common, high activity may be representative of a healthy vaginal microbiome.

A limitation of this exploratory study is the small sample size. Thus, as noted above, we cannot preclude a broader association with other healthy *Lactobacillus species.* Furthermore, conclusions about the effect of race, ethnicity, age, pH, contraceptive use and host immune mediators on the vaginal microbiome should be made with caution. Another study limitation is that we did not confirm sexual abstinence. Finally, differences in the inhibitory activity of bacterial SCS may be impacted by differences in the bacterial yields from which supernatants were obtained. In an effort to account for this difference, we adjusted the *E. coli* cfu by the bacterial cfu/mL used to produce the SCS in a post *hoc* analysis (Figure 3a) or adjusted the SCS by dilution prior to incubating the samples with *E. coli* (Figure 3b).

The current study highlights the possibility that *E. coli* inhibitory activity may be a functional feature of a *Lactobacillus crispatus* dominant healthy vaginal microbiome. In addition to studies with larger more diverse cohorts, future studies should also include proteomic and metabolomic analyses on the sample to further define the nature and origin (host and/or microbiome) of the molecules that contribute to the *E. coli* inhibitory activity. A longitudinal study in pregnancy is ongoing and will help to elucidate whether high inhibitory activity against *E. coli* translates to a reduction in sub-clinical or overt genital tract infection and preterm birth. These findings could promote the identification of novel strategies to enhance the antimicrobial activity of genital tract secretions. Further studies are needed to determine if introducing lactobacilli derived molecules or sustaining a *L. crispatus* dominant microbiome may promote a healthy vaginal environment. These approaches could lead to reduced risk of bacterial vaginosis, chorioamnionitis, preterm birth and perinatal infection and be used as a safer alternative to traditional antibiotics [27].
